# Impact of Organizational Dehumanization on Employee Knowledge Hiding

**DOI:** 10.3389/fpsyg.2022.803905

**Published:** 2022-02-21

**Authors:** Um E. Rubbab, Sana Aroos Khattak, Hina Shahab, Naveed Akhter

**Affiliations:** ^1^Department of Business Administration, Fatima Jinnah Women University, Rawalpindi, Pakistan; ^2^Department of Management Studies, Bahria University, Islamabad, Pakistan; ^3^Department of Management Sciences, National University of Modern Languages, Islamabad, Pakistan

**Keywords:** knowledge hiding, psychological distress, organizational dehumanization, felt obligation for constructive change, conservation of resources theory

## Abstract

Knowledge hiding has become an alarming issue for the organizations. Knowledge hiding is an employee’s intentional attempt to conceal knowledge requested by others at the workplace. Employee knowledge hiding significantly influences an organization’s effective functioning. This research is an attempt to extend previous work on antecedents of knowledge hiding. Drawing on conservation of resources theory, it is proposed that receiving poor treatment by organizations in the form of organizational dehumanization creates psychological distress among employees toward the organization. Distress among workers in turn intervenes the path and increases the likelihood of engaging in knowledge hiding behaviors. An employee’s felt obligation for constructive change (FOCC) may moderate the relationship between organizational dehumanization and employee psychological distress. Data for the current study were collected from 245 employees of the telecommunication sector in three-time lags. The results support the direct and indirect effect of organizational dehumanization on employee knowledge hiding behaviors through the mediation of psychological distress. The results also support the moderation of FOCC between organizational dehumanization and psychological distress. Furthermore, the findings of the study may help organizational practitioners and managers about the value of effective organizational climate and practices for better organizational functioning through knowledge sharing and providing insight into undesirable repercussions of organizational dehumanization. Implications for organizations and practitioners are discussed.

## Introduction

In this era of competition, the organizations are striving to gain a competitive advantage over others by increasing their productivity (Kuranchie-Mensah and Amponsah-Tawiah, 2016). In this competition race, the organizations are pressuring their employees with excessive workloads and mechanical structure while ignoring the humanistic perspective, thus resulting in employee mistreatment. In the past, most of these “negative or abusive” behaviors were attributed to the leadership style of an organizational leader, such as abusive supervision, tyrant leadership, despotic leadership, and perceiving organizations as innocent spectators (Kemper, 2016; Akram et al., 2019; Hussain et al., 2020; Wang et al., 2020; He et al., 2021). Previously some researchers found that an organization can be a source of abuse, hindrance, obstruction, or harm to its employees (Gibney et al., 2009). For example, work overload, lack of organizational support, workplace bullying, lower social support from bosses and peers (Agarwal et al., 2021; Nguyen et al., 2021). The dark side of employee behaviors has emerged as the gravest issue in organizations, depleting employee psychological resources (Irshad and Bashir, 2020; Yao et al., 2020; Pereira and Mohiya, 2021).

When employees perceive their relationship with the organization as harmful and mistreated by the organization, it leads to negative employee and organizational outcomes (Caesens et al., 2017; Morsch et al., 2020; Sarwar and Muhammad, 2020; Wang et al., 2021).

One concept that has recently emerged as destructive for both employees and organizations is organizational dehumanization. Organizational dehumanization refers to the perception of employees about organizational mistreatment as a result of their experience with the organization by treating them like machines rather than humans, having less concern for their respects, and handling them as a means to achieve organizational objectives with less capacity for willingness and sentiments (Caesens and Stinglhamber, 2019; Nguyen et al., 2021; Sainz et al., 2021).

Organizational dehumanization has been studied with the various negative employee and organizational outcomes. For instance, Sarwar and Muhammad (2020) found that organizational dehumanization reduces organizational performance. Further, Sarwar et al. (2021a) stated that organizational dehumanization is the potential predictor of deviant behavior. The adverse outcomes of organizational dehumanization are understandable, and however, employees cannot always reciprocate the mistreatment of the organization overtly. The covert deviant behaviors of employees include time theft, procrastination, and knowledge hiding (Robinson and Bennett, 1995; Webster et al., 2008; Martin et al., 2010; Connelly et al., 2012; Nguyen et al., 2013; Kang, 2016; Prem et al., 2018; Halberstadt et al., 2019). Some studies have made efforts to understand employees’ less dominant but deviant behavior in response to organizational mistreatments such as employee theft and knowledge hiding (Sarwar and Muhammad, 2020). However, they relied on the mediating mechanism of incivility, which is a visible deviant behavior. Sarwar and Muhammad (2020) also recommend testing other explanatory mechanisms between employees’ dehumanization and knowledge hiding.

The current study aims to investigate knowledge hiding as an outcome of organizational dehumanization through the psychological distress of employees. Knowledge hiding refers to the intentional effort of the employees to conceal their personal and professional knowledge and refrain from sharing with others (Connelly et al., 2012; Kang, 2016). Knowledge hiding refers to withholding of information that is related to the task, using delay tactics in sharing information, intentionally concealing information that is organizationally desired, and not sharing implicit knowledge gained through experience (Serenko and Bontis, 2016; Cerne et al., 2017; Connelly et al., 2019). Employees engage in knowledge hiding behaviors to rationalize the ill-treatment of dehumanization and downplay the distress. In response to organizational mistreatment in the form of perceived dehumanization, employees might reciprocate by concealing their knowledge from sharing with others and not sharing innovative achievements. Organizational dehumanization is also a reason for psychological distress in employees. When employees feel that their organization is treating them like robots and has less care for their interests, it creates psychological strain and stress (Robinson et al., 2004; Glicken and Robinson, 2013). Psychological distress is a feeling of emotional discomfort in response to some stressor (Hilton et al., 2008; Ozaki et al., 2012; Sidorchuk et al., 2017; James et al., 2018; Dodia and Parashar, 2020; Viertiö et al., 2021). An employee’s psychological distress is proposed as an explanatory mechanism between the relationship between organizational dehumanization and knowledge hiding of employees.

Further, the personal orientation of employees is a critical factor in deciding employees’ responses to organizational mistreatment. Felt obligation for constructive change (FOCC) refers to the orientation of employees in which they feel responsible for bringing progressive changes in the organization (Phillips-Miller and Morrison, 1999; Fuller et al., 2006). FOCC brings organizations many fruitful outcomes that become the reason for employees’ personal developments and organizational progressions (Fuller et al., 2006; Liang et al., 2012; Mallory et al., 2020). FOCC is used as a valuable resource that is used as a shield against losses experienced by dehumanization practices. As per conservation of resources (COR) theory (Hobfoll et al., 2018), FOCC being a valuable resource will determine individual appraisals of stressful situations and experience less distress if they have high FOCC.

The employees who experience a high level of FOCC appraise the problem with a solution approach rather than an avoidant approach (Liang et al., 2012; Bhatti et al., 2020). Considering the importance of FOCC as a psychological state, it helps as a coping mechanism to deal with psychological distress. Through personal experience, the workers recognize what is important for their sheer survival. The COR theory implies that FOCC will help individuals to replace or restore the loss they have experienced in dehumanization. In short, the COR theory postulates that individuals strive to obtain, retain, foster, and protect those things they centrally value (i.e., resources) (Chen and Fellenz, 2020; Guo et al., 2020). Hence, it is proposed that employees feel an obligation for constructive changes that will reduce the negative effect of organizational dehumanization on psychological distress and ultimately the knowledge hiding behavior of employees.

The COR theory (Hobfoll, 1989) also supports our proposed model. According to the COR theory, employees strive to preserve their resources from stressor or stressful condition, and frequent exposure to stressor causes stress due to loss of valuable resources followed by the defense mechanism of employees to prevent further loss of resources (Holmgreen et al., 2017). Additionally, the COR theory also posits that investing new resources can offset the loss of resources to organizational stressors. Organizational dehumanization acts as a stressor and consumes psychological resources by causing psychological distress. As a result of resource loss to dehumanization, employees use defensive tactics to regain further resources or stop the resources loss cycle. These defensive techniques might be in the form of knowledge hiding behaviors. Further, FOCC is a valuable resource of employees that can be invested to reduce the damages of stressor organizational dehumanization. Thus, employees with high FOCC will be less vulnerable to organizational dehumanization than others due to their additional resources pool.

The current study contributes to the literature in multiple ways. First, knowledge hiding is proposed as a critical but less dominant deviant behavior resulting from organizational dehumanization. Sometimes employees are not able to reciprocate with the same intensity to organizational mistreatments. Second, psychological distress is proposed as a possible mediator in organizational dehumanization. Previous studies have investigated observable behaviors and attitudes as explanatory mechanisms, while psychological distress is employee’s less visible emotional state that might result in less visible behaviors. Third, FOCC has proposed a potential boundary condition to dampen the effect of organizational dehumanization on psychological distress. Last, the current study has extended the implication of COR theory in the organizational mistreatment literature.

## Literature Review

### Organizational Dehumanization and Knowledge Hiding Behavior

When organizations objectify an employee, it thwarts an individual’s personal needs. The employees perceive to become means to achieve organizational goals (Brison et al., 2021; Sainz and Baldissarri, 2021). They feel like a means to meet organizational ends. But such means make them feel like an instrument or nothing less than a robot (Caesens et al., 2017, 2019; Nguyen and Stinglhamber, 2021; Sainz et al., 2021). But at the same time, they may experience cognitive, physical, and emotional strains. The strains deplete resources and are being used to protect what they already have rather than pursuing personal needs. These stressors subsequently damage productive behaviors (Caesens et al., 2017, 2019). So through this mechanism, we are trying to understand the remitting effect of dehumanization. The underlying mechanism of transmitting the effect of organization dehumanization on knowledge hiding can be *via* psychological distress.

The perception of being dehumanized has detrimental effects on different work attitudes and behaviors between the employee–organization relationships (Sainz and Baldissarri, 2021). Humans have been seen as commodities rather than “human capital” (Väyrynen and Laari-Salmela, 2015). To rationalize the ill-treatment of an organization, they behave negatively to downplay the distress, which also has a profound impact on employee psychological wellbeing (Farh and Chen, 2014; Hirschle and Gondim, 2020; Walsh and Arnold, 2020). Individuals may fail to maintain their normative behavior due to loss of self-resources caused by dehumanization (Haslam and Loughnan, 2014). When the reasons for dehumanization become incomprehensible and when the employees cannot cognitively process the motives, they indulge in deviance (Guo et al., 2020; Sarwar et al., 2021b). Work sabotage, showing up late, organizational theft, absenteeism, and disclosing company secrets are examples of counterproductive work behavior (Bennett et al., 2019; Chen et al., 2018; Yasir and Rasli, 2018).

Previous studies found that the individuals will try to cope with the undermining feeling by protecting their limited resources (Connelly et al., 2012; Dahling, 2017; Feng and Wang, 2019). Following this logic, we argue that organizational dehumanization will result in detrimental psychological consequences in the form of psychological distress, which will then instigate sufferers to take knowledge hiding as a form of resource loss preventing actions (Jiang et al., 2019; Bari et al., 2020; Rezwan and Takahashi, 2021). Knowledge hiding is being studied as a dormant form of deviant behavior responding to organizational stressors (Škerlavaj et al., 2018; Livne-Ofer et al., 2019; Khoreva and Wechtler, 2020). Drawing on the COR perspective (Hobfoll, 2001), employees become defensive and indulge in coping strategies.

**H1:** Organizational dehumanization is positively associated with knowledge hiding behavior of employees.

### Mediation of Psychological Distress Between Organizational Dehumanization and Knowledge Hiding Behavior of Employees

Drawing on the proposition that organization dehumanization may encourage knowledge hiding behavior, we postulate that psychological distress mediates the relationship between organizational dehumanization and the knowledge hiding behavior of employees. Individuals feel distressed when high-order need of relatedness and compassion are denied (Bell and Khoury, 2011; Caesens et al., 2017). Based on the COR theory, organization dehumanization is a resource-draining factor (Volpato and Andrighetto, 2015). The feeling of psychological distress becomes high when the perception of dehumanization is internalized. Bell and Khoury (2016) found that the feelings of disrespect, humiliation, and neglect will enhance dehumanization making employees less socially valuable (Christoff, 2014; Huo et al., 2016). Sarwar and Muhammad (2020) explained in their work that such mechanistic dehumanizing experiences may hinder the process of information sharing in the organization and may inculcate knowledge hiding behavior (Zhang and Min, 2021; Zhao and Jiang, 2021). Workers are not in a powerful position to reciprocate similarly, so they take discourse in indulging in deviant behaviors (Foulk et al., 2016). Knowledge hiding is considered a reaction to the organization’s dehumanization by intentionally withholding necessary organizational knowledge (Burmeister et al., 2019; Farooq and Sultana, 2021).

In a situation of psychological distress, likely, a worker may not be able to suffice the request of any knowledge sharing to guard against the already left energy and time (Khoreva and Wechtler, 2020; Rezwan and Takahashi, 2021). Similarly, Vayrynen and Laari-Salmela (2018) found that employees’ perception of dehumanization brings employees into a negative mental state that indicates psychological distress. Despite these findings, we do not know much about how organizational dehumanization might affect knowledge hiding. Given that, dehumanization has devastating effects by creating distress. Therefore, we seek to examine the antecedents of knowledge hiding from organizational dehumanization mainly through the explanatory mechanism of psychological distress.

**H2:** Psychological distress mediates the relationship between organizational dehumanization and knowledge hiding behavior of employees.

### Moderation of Felt Obligation for Constructive Change

Drawing on the COR theory (Hobfoll, 1989, 2011), dehumanization depletes our cognitive resources and may alienate one’s attitude toward undesirable behavior (Ridner, 2004; Lee et al., 2018; Yan et al., 2020). An individual’s disposition to perceive a negative stimulus in the environment influences our reaction to the event (Judge and Larsen, 2001; Wu and Griffin, 2012). One such individual orientation is the FOCC. It is an individual’s orientation in which employees take outstanding intrinsic inspiration from their work and feel responsible for the assigned task (Fredrickson, 2001). Such disposition is considered very prototypical of a progressive mindset (Mossakowski and Zhang, 2014). We argue that FOCC will lessen the perception that the organization dehumanizes them. Therefore, we can postulate that FOCC will convey to employees that they are in charge of their work and can add value and worth, reducing organizational dehumanization perceptions. In summary, FOCC is an individual’s disposition that will ease the dehumanization’s stressful condition (Jahanzeb et al., 2020; Sainz et al., 2021). FOCC is explained as the employees’ personal sense of responsibility for initiating progressive organizational changes (Fuller et al., 2006). FOCC is essential for ensuring employees’ personal development and organizational progression (Mallory et al., 2020). This depicts that FOCC is a valuable personal-psychological resource that could buffer the negative relation between dehumanization and psychological distress. As explained in the COR theory, the employees strive to preserve their resources from stressors or stressful conditions to ensure their well-being and distance themselves from threats to well-being. Frequent exposure to this organizational dehumanization as a stressor causes psychological distress to employees. The COR theory proposed that in such a stressful environment, employees’ defense mechanisms are activated to prevent or buffer the further loss of resources. The defense mechanism in a given scenario is the individual personal resource, which is FOCC, which thwarts the resource loss cycle (Xanthopoulou et al., 2009; Holmgreen et al., 2017; Pignata et al., 2017).

Given that psychological distress plays a critical role in regulating employees’ attitudes and behaviors (Lee et al., 2018), not every person will respond in the same manner when faced with organizational dehumanization (Lebel, 2017; Kittel et al., 2021). FOCC will try to preserve the loss of individual psychological and physical resources (Fuller et al., 2006) by mitigating the negative effects of organizational dehumanization. Having support from self-regulation theory (Bandura, 1991), we expect that FOCC will act as a boundary condition between dehumanization and psychological distress (Liang et al., 2012). Particularly when faced with dehumanization, employees with high FOCC are more likely to interpret such situations as less intense (Abbasi et al., 2021). They will perceive themselves to be in control of themselves, having proactive conceptualization of the problem (Li et al., 2020) and will experience less resource drain compared with those who have low FOCC. The proactive aspect of personality becomes a protective cover against the stressors in the environment (Bajaba et al., 2021; Wei et al., 2021). They can appraise the situation more optimistically, thus promoting adaptive behaviors (Mazzetti et al., 2019a). Control is one of the characteristics of a hardy personality that considers change as desirable and natural (Mazzetti et al., 2019b).

**H3:** Felt obligation for constructive changes moderates the relationship between organizational dehumanization and psychological distress, such that the relationship will be weaker when FOCC is high and stronger when FOCC is low.

**FIGURE 1:**
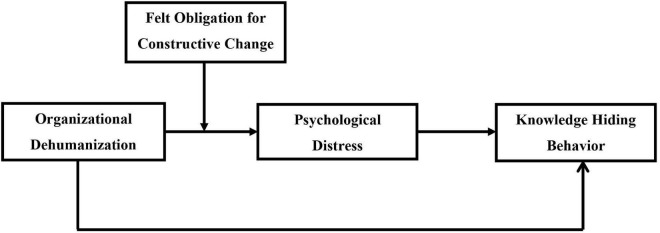
Proposed hypothesized model.

Figure 1 shows the proposed model.

## Materials and Methods

### Sample and Procedure

A self-survey was administered in the service industry, particularly in the telecommunication sector. Concern for knowledge management among employees of the telecom sector is relatively high (Jyoti et al., 2011; Jyoti and Rani, 2017). Increasing demands for innovation and a global knowledge-based economy have fostered the telecom sector to understand that knowledge management can be the real asset to remain competitive and ahead of competitors (Yen et al., 2021). The knowledge database must be maintained by IT specialists so that tasks can be performed effectively (Bender and Fish, 2000). The sustainability and competitive edge of the telecom sector is highly dependent on knowledge management (Alavi and Leidner, 2001). It has also been proposed by Fey and Furu (2008) that competitive advantage is derived from leveraging knowledge. In a study by Wang and Noe (2010), it was reported that 90% of respondents from the telecom sector intended to conduct a study on knowledge management. Therefore, data were taken from employees of the telecom sector. To avoid the common method bias (Podsakoff et al., 2012), which is possible in survey studies (Podsakoff et al., 2003), certain steps at the design stage were ensured, and data were collected in time lags.

Data on predictor variables, that is, organizational dehumanization and moderator variables that are felt as obligations for constructive change, were collected at time lag 1 (T1) at the start of February 2021. Being a time lag study, response on mediator variable, that is, psychological distress, was collected in mid of March 2021. Response on the dependent variable as knowledge hiding behavior was collected in time lag 3 at the start of April 2021. Data collection on all the variables from three-time lags was completed at the end of April 2021. Convenience sampling, a non-random sampling technique, was used for data collection. The size of the sample was determined by G*Power version 3.1.9.4 (Faul et al., 2009). *A priori* sample size was calculated as 107, which is much lesser than the current study sample, that is, 245. This technique is being used by most recent studies and getting the attention of researchers (Irshad et al., 2021b; Qasim et al., 2021). Data were collected through visiting the various organization in an offline mode. Approval was taken from Human Resource offices of telecom organizations to contact their employees. A cover letter explaining the study details was attached to the questionnaires, and employees were assured of their anonymity. Some employees were interested in knowing our study’s findings, so they were assured that findings would be shared with them too. An email address of the corresponding author was mentioned on the cover letter provided along with the questionnaire. So if the respondents are interested in inquiring, they can easily contact us through the corresponding email address. All measures were in English as this is the official language of Pakistan and used the medium for conveying the knowledge in schools and universities. Previous studies have also used English for conducting surveys and administering questionnaires (Um-e-Rubbab and Naqvi, 2020; Irshad et al., 2021a; Majeed et al., 2021).

At T1, 500 questionnaires were distributed and 462 were returned with a response rate of 92%. After a gap of 1 month, the same employees were contacted again and were required to respond on knowledge hiding for T2. At T2, 370 questionnaires were received back. At T3, response on felt obligation was collected from the same employees, and 305 questionnaires were received. Thirty-eight questionnaires were discarded because they were incomplete. So the final response rate was 48% with 267 well-filled questionnaires. Out of the 245 respondents, 144 were male and 101 respondents were female. A total of 71% of respondents were between 21 and 40 years old, 80% had bachelors or more than bachelors degree, 44% had more than 5 years experience, 22% had an experience of 3–5 years, 13% had an experience of 1–3 years, while the remaining had less than 1 year experience (see Table 1).

**TABLE 1:** Respondent characteristics.

Variable	Frequency	Percentage
**Gender**		
Male	144	59
Female	101	41
**Age**		
21–30 years	72	29
31–40 years	103	42
41–50 years	43	18
50 and above	27	11
**Education**		
Below bachelor	48	20
Bachelor	70	28
Masters and above	127	52
**Experience**		
Less than 1 year	51	21
1–3 years	32	13
3–5 years	53	22
5–7 years	69	28
7 and above	40	16

*N = 245.*

### Measures

The scales of perceived organizational dehumanization, perceived distress, knowledge hiding, and felt obligation were adopted from previous studies as mentioned below.

### Perceived Organizational Dehumanization

To measure the employees’ perception of organizational dehumanization, 11-items scale of Caesens et al. (2017) was used. Sample items include “My organization treats me as if I were a robot.” and “My organization considers me as a number.” The respondents were asked to respond on a 5-point Likert scale with 1 for strongly disagree and 5 for strongly agree.

### Psychological Distress

A 10-items scale was adopted from Kessler et al. (2003) to measure the psychological distress of employees. Specifically, the employees were provided with a series of statements like “In the past 4 weeks, about how often did you feel tired out for no good reason? 2. In the past 4 weeks, about how often did you feel nervous?” on a 5-point Likert scale with 1 for none of the time and 5 for all of the time.

### Felt Obligation for Constructive Change

A seven-items scale adapted from the Eisenberger et al. (2001) was used. Sample item includes “I owe it to the organization to do whatever I can to come up with ideas/solutions to achieve its goals.” Employees rated a five-point scale on a 5-point Likert scale with 1 for strongly disagree and 5 for strongly agree.

### Knowledge Hiding

A three-items scale adopted from Peng (2013) was used to measure knowledge hiding. Employees were provided with statements like “Do not want to transform personal knowledge and experience into organizational knowledge” and “Do not share innovative achievements” on a 5-point Likert scale with 1 for strongly disagree and 5 for strongly agree.

## Results

### Correlation Analysis

Table 2 provides the mean, SDs, reliabilities, and correlations among the study variables. An ANOVA was performed to check the variance in perceived distress and knowledge hiding due to demographic variables, that is, gender, age, education, and experience of respondents. Variance accounted for all demographic variables. Independent variables were found to be non-significant. Hence, all the demographic variables were not controlled in the study and were excluded in further analysis. Perceived organizational dehumanization is significantly correlated with perceived distress (*r* = 0.34^**^, *p* < 0.01) and employee knowledge hiding (*r* = 0.49^**^, *p* < 0.01). The FOCC was found to be significantly correlated with perceived distress (*r* = −0.32^**^, *p* < 0.01) and employee knowledge hiding (*r* = −0.26*, *p* < 0.01). Knowledge hiding was significantly correlated with perceived distress (*r* = 0.45^**^, *p* < 0.01).

**TABLE 2:** Mean, standard deviation, reliability, and correlation.

S. No.	Variable	*M*	SD	α	1	2	3	4
1.	Organizational dehumanization	3.20	0.81	0.89				
2.	Psychological distress	3.31	0.87	0.91	0.34**			
3.	Knowledge hiding	3.34	0.89	0.74	0.49**	0.45**		
4.	Felt obligations for constructive change	3.19	0.93	0.89	−0.24**	−0.32**	−0.26**	

S. No., serial number; M, mean; SD, standard deviation; α, reliability. N = 245; **p < 0.01.

### Hypothesis Testing

Table 3 provides the direct, mediation, and moderation hypotheses. Hayes (2017) Model 4 of the PROCESS macro was used to check the mediation, and Model 1 was used to check the moderation hypothesis. In line with Hypothesis 1, perceived organization dehumanization was significantly associated with knowledge hiding (β = 0.41, *p* < 0.01); thus, the H1 of the study was accepted. Furthermore, perceived organizational dehumanization was significantly associated with perceived distress (β = 0.37, *p* < 0.01), and perceived distress was significantly associated with knowledge hiding (β = 0.32, *p* < 0.01). The indirect effects confirm the significant mediating role of perceived distress in the relationship between perceived organizational dehumanization and knowledge hiding [indirect effect = 0.12, 95% CI with lower limit (LL) = 0.07 and upper limit (UL) = 0.18]. The LL and UL of the 95% CI both contain non-zero values (Hair et al., 2014). Hence, H2 is also accepted.

**TABLE 3:** Bootstrapping results for direct and indirect effects.

Direct effects		Effect	SE		*t*
H1	Organizational dehumanization → knowledge hiding	0.41**	0.06		6.71
	Organizational dehumanization → psychological distress	0.37**	0.06		5.71
	Psychological distress → knowledge hiding	0.32**	0.05		5.67

**(95% bias corrected confidence interval method)**					

**Indirect effects**		**Effect**	**SE**	**LL**	**UL**

H2	Organizational dehumanization → psychological distress → knowledge hiding	0.12	0.03	0.07	0.18

LL, lower limit; UL, upper limit; SE, standard error. N = 245, **p < 0.01.

**FIGURE 2:**
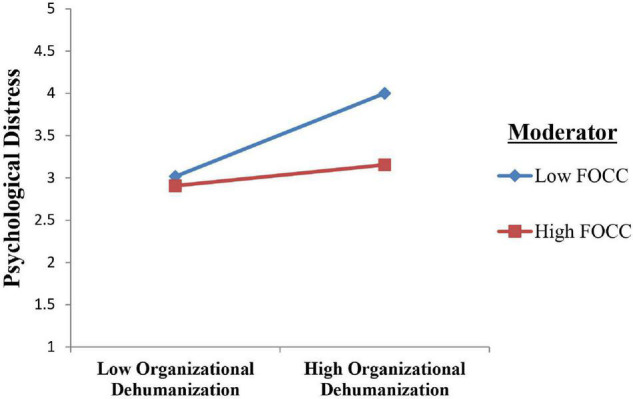
Felt obligation for constructive change (FOCC) dampens the positive relationship between organizational dehumanization and psychological distress.

Table 4 presents the result for moderation analysis. Before testing Hypothesis 3, perceived organizational dehumanization and FOCC were mean-centered (Aiken et al., 1991). The interaction effect of perceived organizational dehumanization and FOCC was significant (β = −0.18, *p* < 0.01). Hence, H3 is also supported. Table 4 and Figure 2 also show the conditional effect of organizational dehumanization on psychological distress *via* FOCC getting weaker at high values of FOCC (±1 SD from the mean).

**TABLE 4:** Moderation analysis.

Felt obligation for constructive change				

		β	SE	Δ*R*^2^
	Constant	3.27		
	Organizational dehumanization → psychological distress	0.31**	0.06	
	Felt obligation for constructive change → psychological distress	−0.24**	0.05	
**H3**	Organizational dehumanization × felt obligation for constructive change → psychological distress	−0.18**	0.07	0.022**

**Conditional effects of moderator at *M* ± 1 SD (slope test)**	**Effect**	**SE**	**LL 95% CI**	**UL 95% CI**

Felt obligation for constructive change low −1 SD (−0.93)	0.48	0.09	0.30	0.66
Felt obligation for constructive change *M* (0.00)	0.30	0.06	0.18	0.43
Felt obligation for constructive change +1 SD (0.93)	0.13	0.08	−0.03	0.31

LL, lower limit; UL, upper limit; CI, confidence interval; SD, standard deviation; M, mean; SE, standard error. N = 245, p** < 0.01.

## Discussion

In the age of global pandemic, the organization’s sustainability has become the subject of attention as it has led to fierce competition for survival and productivity (Al Aina and Atan, 2020; Keshky et al., 2020; Ibn-Mohammed et al., 2021). In this crisis time, the organizations are mostly shifting toward resource optimization strategies; they end up falling for opting for mechanistic culture and styles of leadership rather than choosing humanistic cultures. Organizations invest a lot in knowledge management and offer many incentives to promote knowledge at various levels, from employees at the same level and from subordinates to managers and *vice versa*. Hence, employees do not share knowledge and try to hold information despite the organizational efforts (Connelly et al., 2012).

Our study findings suggest that one reason for all efforts and resources spent on making employees share the knowledge go in vain can be the certain organizational practices and behaviors. When employees perceive that they are being treated as robots by the organization, they start acting like robots reciprocating the organizational maltreatment. The maltreatment of employees by the organization is termed as organizational dehumanization. Organizational dehumanization kills employees’ connectedness and belongingness to the organization, and employees prefer hiding their knowledge to justify organizational ill-treatment. Organizational dehumanization serves as a stressor and tends to drain employees psychologically. Since knowledge is an important resource and human beings strive to conserve and retain valuable resources (Hobfoll, 1989). According to Hobfoll (1989), when an individual perceives that an external stressor threatens his/her resources (e.g., psychological, social, and physical), he/she would try to protect and conserve his/her valuables by engaging in certain behaviors. In the current scenario, employee knowledge hiding is his/her retaliatory behavior in response to organizational dehumanization. When employees perceive ill-treatment by the organization, being abused or being used as objects by the organization, they start engaging in retaliation (Khalid et al., 2018), considering their knowledge as a tool to conserve and hold.

Glancing from the COR theory perspective, it proposes that the prevalence of such dehumanizing cultures can create psychological distress or strain among the employees and abstain from further psychological distress and personal resource depletion; the employees mostly exhibit defensive behaviors on their job, such as knowledge hiding. The theory also postulates that the employees can buffer this negative relationship between job demands and psychological strains if they carry certain personal resources to alleviate themselves from this vicious resource depletion cycle (Holmgreen et al., 2017).

This study proves that dehumanizing cultures positively related to increasing employees’ distress, which further increases knowledge hiding behaviors among employees. This study also contributes to the existing literature and theory by investigating the moderating role of the FOCC by weakening the strong relationship between organizational dehumanization and employee distress. The study findings also showed the negative relationship between FOCC and employee psychological distress. FOCC is explained as the employee’s personal sense of responsibility for initiating progressive organizational changes (Fuller et al., 2006). The COR theory proposed that employees’ defense mechanisms are activated in such a stressful environment to prevent or buffer further loss of resources. The defense mechanism in a given scenario is the individual personal resource, which is FOCC, which thwarts the resource loss cycle (Xanthopoulou et al., 2009; Holmgreen et al., 2017; Pignata et al., 2017).

This current study offers several insights and guidelines for practitioners. First, it highlights that any organization’s sustainability, competition, and productivity can never reside in shifting toward mechanistic cultures; instead, it requires innovative cultures that premise upon knowledge-sharing cultures. Such short-term thinking of dehumanizing organizations such as abusive supervision could worsen the organization and ignite vicious cycles of resource depletion among the organizations.

Second, the dehumanization cultures are strongly related to creating employees’ psychological discomfort and distress. Studies already show that distressed employees can never be engaged in productive outputs like innovation (Tepper et al., 2007; Park et al., 2018). Third, this study also provides evidence that distressed employees end up safeguarding themselves by engaging in defensive behaviors such as knowledge hiding, which can destroy the spirit of creativity and innovation and diminish any organization’s future growth and survival.

Last, this study also provides contextual solutions and insight to organizations that have mechanistic cultures or have high job demands structures; they should recruit employees who have a strong sense of responsibility or feel an obligation toward change because such individuals have such strong aspirations that despite having high job pressures or negative work cultures their inner state of self-responsibility does not get them effected by these job constraints. Hence, they do not feel distressed about their job. This study can also lead to new directions that individuals having felt an obligation for change could effectively redirect the dehumanizing organization toward positive humanistic cultures. Future studies could also identify other moderators like self-efficacy, organizational commitment, and some personality characteristics that could help to buffer the high job demand constraints on employees.

### Research Limitations and Future Directions

This study provides evident theoretical and methodological contributions; however, it also carries certain limitations. First, this study observed the impact of organizational dehumanization and psychological distress on only one dependent variable: knowledge hiding behavior. Future studies could also include other counterproductive work behaviors, such as an important outcome in the form of employee procrastination can be studied as the possible outcome of organization dehumanization too. Second, FOCC was taken as a personal psychological resource.

In contrast, other personal resources like resilience, hope, and psychological capital or certain personality traits can also be studied as possible strengthening and buffering effects. This study followed a time-lagged methodology for data collection, and future researches should consider longitudinal studies in other sectors and different contexts.

## Conclusion

The current study focuses on the effects of dehumanization practices in the telecommunication sector, although lacking extreme symbolic violence. But other factors lie under the surface. Certain practices are invisible and difficult to change. As a whole, the result of our study suggests that organizational dehumanization should not be left unchecked. Otherwise, it can create a stressful environment damaging employees’ mental state. It may lead to deviant behavior in the form of knowledge hiding. The violation of basic humanness is detrimental for individuals and can also have adverse effects for organizations by the intentional act of concealing vital information. Environmental influence or individual disposition (FOCC) reduces the feeling of being treated like an instrument or tool.

## Data Availability Statement

The original contributions presented in the study are included in the article/supplementary material, further inquiries can be directed to the corresponding author.

## Ethics Statement

The studies involving human participants were reviewed and approved by the FJWU Ethics Committee. The patients/participants provided their written informed consent to participate in this study.

## Author Contributions

All authors listed have made a substantial, direct, and intellectual contribution to the work, and approved it for publication.

## Conflict of Interest

The authors declare that the research was conducted in the absence of any commercial or financial relationships that could be construed as a potential conflict of interest.

## Publisher’s Note

All claims expressed in this article are solely those of the authors and do not necessarily represent those of their affiliated organizations, or those of the publisher, the editors and the reviewers. Any product that may be evaluated in this article, or claim that may be made by its manufacturer, is not guaranteed or endorsed by the publisher.
